# The Interaction of TPH2 and 5-HT2A Polymorphisms on Major Depressive Disorder Susceptibility in a Chinese Han Population: A Case-Control Study

**DOI:** 10.3389/fpsyt.2019.00172

**Published:** 2019-04-04

**Authors:** Jiarun Yang, Xueyan Zhao, Jingsong Ma, Zhengxue Qiao, Xiuxian Yang, Erying Zhao, Bo Ban, Xiongzhao Zhu, Depin Cao, Yanjie Yang, Xiaohui Qiu

**Affiliations:** ^1^Department of Medical Psychology, Institute of Public Health, Harbin Medical University, Harbin, China; ^2^Department of Endocrinology, Affiliated Hosptial of Jining Medical University, Jining, China; ^3^Medical Psychological, Institute of the Second Xiangya Hospital of Central South University, Changsha, China; ^4^Department of Medical Education Management, Harbin Medical University, Harbin, China

**Keywords:** major depressive disorder, TPH2, 5-HT2A, gene-gene interaction, polymorphisms

## Abstract

**Purpose:** TPH2 and 5-HT2A appear to play vital roles in the homeostatic regulation of serotonin levels in the brain, their genetic variations may lead to impaired homeostatic regulation of serotonin resulting in abnormal levels of serotonin in the brain, thus predisposing individuals to MDD. However, research studies have yet to confirm which gene-gene interaction effect between TPH2 and 5-HT2A polymorphisms results in increased susceptibility to MDD.

**Methods:** A total of 565 participants, consisting of 278 MDD patients and 287 healthy controls from the Chinese Han population, were recruited for the present study. Six single nucleotide polymorphisms (SNPs) of TPH2/5-HT2A were selected to assess their interaction by use of a generalized multifactor dimensionality reduction method.

**Results:** A-allele carriers of rs11178997 and rs120074175 were more likely to suffer from MDD than T-allele carriers of rs11178997, or G-allele carriers of rs120074175. The interaction between TPH2 (rs120074175, rs11178997) and 5-HT2A (rs7997012) was considered as the best multi-locus model upon the MDD susceptibility.

**Conclusions:** Our data identified an important effect of TPH2 genetic variants (rs11178997 and rs120074175) upon the risk of MDD, and suggested that the interaction of TPH2/5-HT2A polymorphism variants confer a greater susceptibility to MDD in Chinese Han population.

## Introduction

Major depressive disorder (MDD) is a frequently occurring mental disorder with a strikingly high rate of relapses, and has become the leading cause of years lived with disability worldwide according to data compiled by the World Health Organization. Patients suffering with MDD struggle with severe role impairment (1) and most suffer from suicidal ideation, suicidal intent or suicidal attempts (2). Genetic factors have been found to be an important contributor to MDD (3), and inheritance can increase the risk of developing MDD by 40–70% (4).

Previous studies have provided strong evidence that serotonergic transmission is altered in MDD (5), and have identified polymorphism variants that are associated with deficits in the transmission of serotonin, which are believed to be involved in the pathophysiology of MDD (6). Polymorphism of the tryptophan hydroxylase-2 (TPH2) gene is one such example. TPH2 is the rate-limiting enzyme for the synthesis of serotonin in the brain (7). Functional polymorphisms of the TPH2 gene have been found to affect serotonin synthesis capacity and serotonin neurotransmission (8, 9), and several TPH2 gene polymorphisms have been confirmed to be associated with MDD. The first report of the association between TPH2 and MDD was published by Zill et al. (10). Subsequently, rs120074175 (1463G/A), a functional polymorphism variant of TPH2, which was shown to attenuate the synthesis of serotonin by ~80%, was also associated with MDD (8). A meta-analysis also showed that TPH2 rs4570625 (-703G/T) has strong epidemiological credibility for an involvement with MDD (11). In addition, the association of MDD and a haplotype block including rs4570625 and rs11178997 (473T/A) was confirmed (12). Collectively, these findings strongly indicated that TPH2 polymorphisms may play an important role in the development of MDD. In spite of the fact that TPH2 polymorphisms could result in the attenuation of serotonin synthesis, the genetic variation of TPH2 in isolation is unlikely to lead to a high risk of MDD (13, 14).

Interestingly, one research study suggested that the risk imposed by TPH2 may be modulated by the serotonin 2A receptor (5-HT2A) (15), an important regulator of the serotonin signaling (16). Multiple animal studies have confirmed that 5-HT2A binding is associated with serotonin levels (17, 18). In addition, changes in 5-HT2A binding levels have also been identified in different regions of the brain in depressed individuals (19, 20). Some genetic studies have also shown the association of 5-HT2A polymorphisms and MDD; for example, rs6311, rs6313, rs7997012 (21–23). 5-HT2A variants were identified as showing specific associations with MDD.

MDD is a polygenic disease; in other words, there are multiple and partially overlapping sets of MDD susceptibility genes which interact with each other, thus predisposing individuals to the development of MDD (24). Over recent years, genetic studies have identified numerous genetic variants implicated in MDD. However, results arising from the analyses of single markers have often been inconsistent, and for many candidate SNPS, results could not be replicated (25). Gene–gene interaction analysis is a promising method that can reveal susceptibility genes and their interaction, and has been confirmed as a particularly important method for revealing the molecular mechanisms of complex human diseases, such as MDD (26).

Based on the earlier observations, TPH2 and 5-HT2A appear to play vital roles in the homeostatic regulation of serotonin levels in the brain. Furthermore, TPH2 and 5-HT2A genetic variations may lead to impaired homeostatic regulation of serotonin resulting in abnormal levels of serotonin in the brain for long periods of time, thus predisposing individuals to MDD. However, research studies have yet to confirm what gene–gene interaction effect between TPH2 and 5-HT2A results in increased susceptibility to MDD.

The purpose of the present study was thus to investigate the specific interaction between the TPH2 and 5-HT2A genes and whether this mechanism contributes to susceptibility for MDD. SNPs selected in this study are not only associated with MDD but also associated with its gene expression (8, 27–29). Resultant data will contribute to a better understanding of MDD and genetic predisposition, and will assist in further interpreting the role of serotonin in susceptibility for MDD.

## Materials and Methods

### Subjects

In total, 278 MDD patients, and 287 healthy controls, were recruited for the present study. All subjects were from the Chinese Han population, and were living in the same geographical area in the north of China. MDD patients were diagnosed according to the criteria for MDD described in the Fourth Edition of Diagnostic and Statistical Manual of Mental Disorders (DSM-IV). The 24-item Hamilton Rating Scale for Depression (HAMD) was used to evaluate all patients. Our inclusion criteria required that subjects had a HAMD score of 21 or higher, and had not received antidepressant treatment for 1 month preceding assessment. Our exclusion criteria included: (1) a history of brain organic mental disorders or other mental disorders; (2) a family history of genetic disorders; (3) individuals with mental retardation or dementia; and (4) individuals recently receiving blood transfusion treatment. All psychiatrists involved in patient diagnosis were specifically trained using the Structured Clinical Interview for DSM-IV disorders (SCID-I). Each patient was interviewed independently by at least two psychiatrists. Written informed consent was obtained from all participants and the study was approved by the Research Ethics Committee of Harbin Medical University, China.

### DNA Isolation and Genotyping

Venous blood was taken from all participants and DNA isolated from EDTA-anticoagulated blood samples using the AxyPrep^TM^ Blood Genomic DNA Minprep Kit (Axygen, Union City, CA, USA). Primer Premier 5.0 software was used to design the primers for polymerase chain reaction (PCR) amplification and final primers were evaluated using NCBI-BLAST (https://blast.ncbi.nlm.nih.gov/Blast.cgi). Primer sequences are given in Table 1.

**Table 1:** The primers sequence for SNPs of TPH2 and 5-HT2A.

**Gene**	**SNP ID**	**Polymorphisms**	**Location**	**Primer sequence (5^**′**^ → 3^**′**^)**
TPH2	rs4570625	G/T	5′Regulatory region	F: 5′-AGTAGAGAGAAAAACCACAAGAGTATT-3′ R: 5′-CATTGCCTCAAGCATTTATCA-3′
	rs11178997	A/T	5′Regulatory region	F: 5′-TCCTTTTATCTATCCCTCGTACCAA-3′ R: 5′-GGTCCTGCACCACATTTTCA-3′
	rs120074175	A/G	Coding region	F: 5′-TGAGGCAATGGATATCTTGATTACC-3′ R: 5′-GCACTGCTGAATGCTTAAACCA-3′
5-HT2A	rs7997012	A/G	Intron region	F: 5′-CTAACCTCATGTCACCTCACA-3′ R: 5′-ATGTTGCAAACAATGGGCCAG-3′
	rs6311	T/C	5′Regulatory region	F: 5′-ATGGCCTTTTGTGCAGATTCC-3′ R: 5′-AGAGTTATCACCACAGACGTG-3′
	rs6313	T/C	Intron region	F: 5′-GCTACAAGTTCTGGCTTAGAC-3′ R: 5′-TGAAGTAAGGAGAGACACGAC-3′

DNA samples (TPH2: rs4570625, rs120074175, rs11178997; 5-HT2A: rs7997012, rs6311, rs6313) were genotyped using PCR TaqMan assays and read on an ABI PRISM 7900 Sequence Detection System (Applied Biosystems, Foster City, CA, USA). PCR amplicons were sequenced and purified using an ABI 3730 DNA Sequencer.

### Statistical Analysis

The Hardy–Weinberg equilibrium test was carried out for all SNP loci and an independent-samples *t*-test was used to estimate differences in age distribution between case and control groups. The chi-square test was used to investigate differences in categorical variables, such as gender or differences in the distribution of genotype. A *p* < 0.05(two-tailed) was considered as statistically significant. SPSS 19.0 software was used for all statistical analysis.

Haplotype analysis was performed using Haploview software (version 4.2) and analyzed differences in haplotype frequencies between case and control groups. Gene-gene interactions were tested using generalized multifactor dimensionality reduction (GMDR) software (version v0.7) and default parameters. In the configuration file, 10-fold cross-validation (CV) was defined and threshold ratio set at 1.0. The analysis was performed ten times using ten different random number seeds. The results were averaged to avoid spurious outcomes due to chance division of the data. Age and gender were regarded as covariates. The best gene–gene interaction model was selected based upon the values arising from CV consistency and accuracy testing. Interaction graphs and dendrograms were used to help interpret a multi-locus model of disease susceptibility.

## Results

Participant characteristics are shown in Table 2. In total, our study incorporated 565 participants, including 278 MDD patients and 287 healthy controls. There were 74 males (26.6%) and 204 females (73.4%) in the MDD group, and 83 males (28.9%) and 204 females (71.1%) in the control group. The mean age of MDD cases and controls was 42.79 years and 41.90 years, respectively. There was no statistically significant difference between cases and controls in terms of either gender or age distribution (gender: *p* = 0.378, age: *p* = 0.542). The HAMD score of the MDD group ranged from 21 to 58 points.

**Table 2:** The characteristics of participants.

**Variables**	**MDD (*n* = 278)**	**Control (*n* = 287)**	***p*-value**
Sex(males/females)	74/204	83/204	0.542
Age(mean ± SD)	42.79 ± 12.21	41.90 ± 11.93	0.378
HAMD score(mean ± SD)	30.38 ± 6.58	5.93 ± 5.76	

The genotype distributions of TPH2 and 5-HT2A polymorphisms conformed to the Hardy–Weinberg equilibrium and the results of our single marker analysis are presented in Table 3. Significant differences in genotypic and allelic distributions between cases and controls were confirmed at locus rs11178997 (*p* = 0.000 for both genotype and allele) and rs120074175 (*p* = 0.000 for both genotype and allele) of the TPH2 gene; after Bonferroni correction, these differences remained significant. Odds ratio analysis showed that A-allele carriers of rs11178997 and rs120074175 were more likely to suffer from MDD than T-allele carriers of rs11178997, or G-allele carriers of rs120074175 (rs11178997: OR = 1.637, 95%CI: 1.290–2.078; rs120074175: OR = 7.909, 95%CI: 5.987–10.447). Differences in the distribution of rs6311 and rs6313 of the 5-HT2A gene were observed only in terms of genotype (rs6311: *p* = 0.029; rs6313: *p* = 0.049), but after Bonferroni correction, these differences were not significant. We did not identify an association between MDD and the other SNPs tested (TPH2:rs4570625, 5-HT2A: rs7997012).

**Table 3:** Genotypic and allelic distributions of TPH2 and 5-HT2A polymorphisms of MDD patients and controls.

**Gene**	**SNP**	**Sample**	**Genotype (%)**			***p***	**Allele (%)**		***p***	**Odds ratio (95%CI)**
TPH2	rs4570625		GG	TG	TT		G	T		
		Case	73 (26.3)	131 (47.1)	74 (26.6)	0.129	277 (49.8)	279 (50.2)	0.079	1.233
		Control	55 (19.2)	146 (50.9)	86 (29.9)		256 (44.6)	318 (55.4)		(0.976–1.558)
	rs11178997		AA	AT	TT		A	G		
		Case	93 (33.5)	170 (61.1)	15 (5.4)	0.000^*^	356 (64.0)	200 (36.0)	0.000^*^	1.637
		Control	135 (47.0)	29 (10.1)	123 (42.9)		299 (52.1)	275 (47.9)		(1.290–2.078)
	rs120074175		AA	AG	GG		A	T		
		Case	207 (74.5)	48 (17.2)	23 (8.3)	0.000^*^	462 (83.1)	94 (16.9)	0.000^*^	7.909
		Control	108 (37.6)	4 (1.4)	175 (61.0)		220 (38.3)	354 (61.7)		(5.987–10.447)
5-HT2A	rs7997012		AA	AG	GG		A	G		
		Case	22 (7.9)	106 (38.1)	150 (54.0)	0.247	150 (27.0)	406 (73.0)	0.257	1.167
		Control	13 (4.5)	112 (39.0)	162 (56.5)		138 (24.0)	436 (76.0)		(0.893–1.526)
	rs6311		TT	CT	CC		T	C		
		Case	82 (29.5)	128 (46.0)	68 (24.5)	**0.029**	292 (52.5)	264 (47.5)	0.662	1.053
		Control	65 (22.6)	164 (57.2)	58 (20.2)		294 (51.2)	280 (48.8)		(0.834–1.330)
	rs6313		TT	CT	CC		T	C		
		Case	79 (28.4)	129 (46.4)	70 (25.2)	**0.049**	287 (51.6)	269 (48.4)	0.847	1.023
		Control	65 (22.6)	163 (56.8)	59 (20.6)		293 (51.0)	281 (49.0)		(0.810–1.292)

*Bolded indicates statistically significant (p < 0.05)*.

* *indicates the difference was still significant after Bonferroni correction*.

Haplotype frequencies in case and control groups were estimated by Haploview software (version 4.2) and our results are shown in Table 4. Strong Linkage Disequilibrium (LD) was observed between the 5-HT2A rs6311 and rs6313 (D′ = 1.0, *r*^2^ = 0.979), but the 5-HT2A rs6311-rs6313 haplotypes were not associated with MDD.

**Table 4:** Haplotype-based association analysis results.

**Gene**	**Haplotype**	**Case ratios**	**Control ratios**	***χ^2^***	***p***
5-HT2A	TT	0.516	0.510	0.037	0.847
	CC	0.475	0.488	0.191	0.662

GMDR was used to detect the interaction effects of six TPH2/5-HT2A SNPs in MDD susceptibility. The results of gene-gene interaction models, using age and gender as covariates, are given in Table 5. In total, 1,000 replications were used to determine the empirical *p*-value of prediction accuracy using the permutation test. Significant 2-locus to 6-locus gene-gene interaction models were observed (*p* < 0.001). The interaction between TPH2 (rs120074175, rs11178997) and 5-HT2A (rs7997012) showed a CV consistency of 10/10 and a testing accuracy of 81.86%, and this was therefore considered as the best multi-locus model. The testing accuracy of all multi-locus models was higher than the accuracy of the best single locus model. This result suggested there was an interaction effect of TPH2 and 5-HT2A upon MDD susceptibility.

**Table 5:** The best gene-gene interaction models obtained by GMDR.

**Locus no**.	**Best model**	**Testing accuracy (%)**	**CV consistency**	***p* value**
1	TPH2(rs120074175)	74.91	9/10	<0.001
2	TPH2(rs120074175,rs11178997)	81.18	10/10	<0.001
3	TPH2(rs120074175,rs11178997), 5-HT2A(rs7997012)	81.86	10/10	<0.001
4	TPH2(rs4570625,rs120074175,rs11178997), 5-HT2A(rs6311)	77.10	5/10	<0.001
5	TPH2(rs4570625,rs120074175,rs11178997), 5-HT2A(rs7997012, rs6311)	77.93	10/10	<0.001
6	TPH2(rs4570625,rs120074175,rs11178997), 5-HT2A(rs7997012, rs6311,rs6313)	77.90	10/10	<0.001

**Figure 1:**
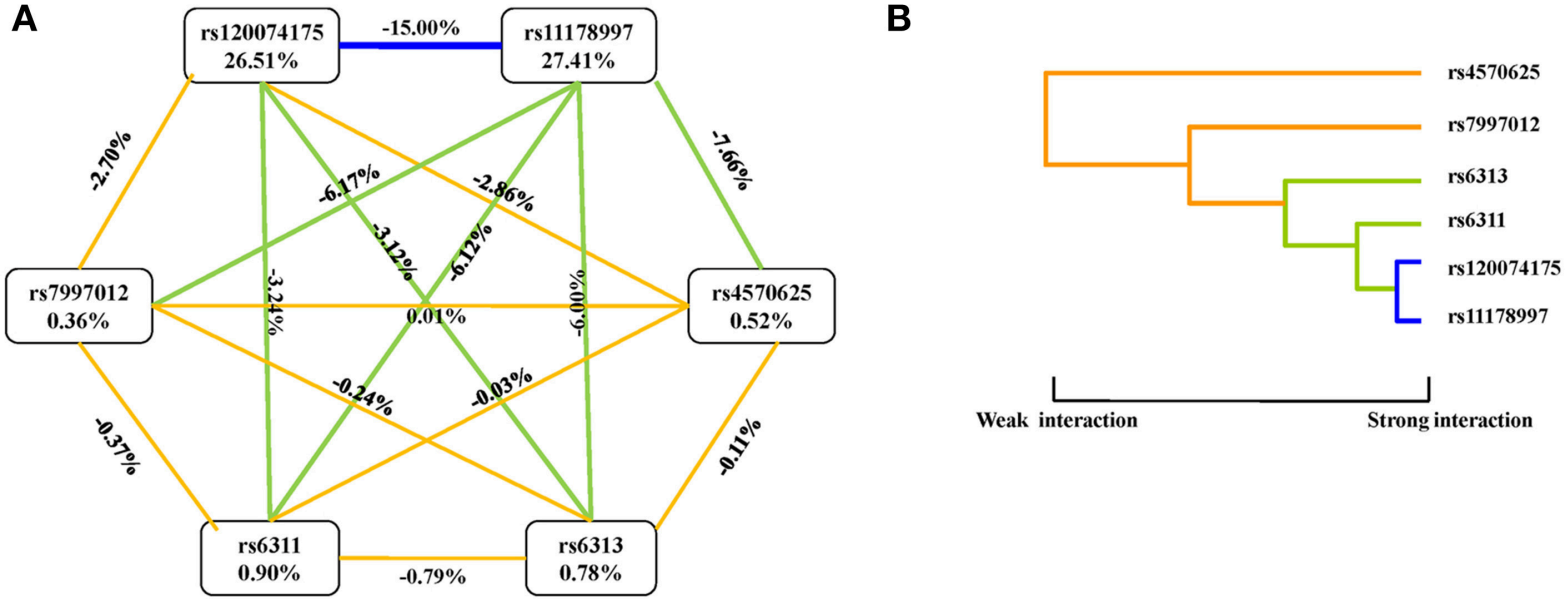
Interaction graphs and dendrograms. **(A)** Interaction graphs. The percentage at the bottom of each SNP represents its interaction entropy, and the percentage on the line represents the percentage of interaction entropy between two SNPs. The golden line indicates additivity, while the blue or green lines indicate redundancy interaction. **(B)** Interaction dendrograms. The golden line represents additivity, while the blue line represents redundancy interaction. The shorter the dendrograms arm, the stronger the interaction.

An interaction graph and an interaction dendrogram were created in order to interpret the relationship between the six SNPs tested. As shown in Figure 1, we found that rs11178997 and rs120074175 both had strong independent effects (rs11178997, 27.41%; rs120074175, 26.51%). The strongest redundant interaction occurred between rs120074175 and rs11178997 in the TPH2 gene with an entropy of−15.00%. Rs120074175 also had a redundant interaction with rs6311 andrs6313, while rs11178997 had a redundant interaction with the other four SNPs. The interactions between other SNPs were relatively independent.

## Discussion

Serotonin has been proven to be involved in the pathogenesis of MDD (6), although the precise role of the serotonin system in MDD is still under debate (5, 30). TPH2 and 5-HT2A are key regulators of central serotonin transmission, and are considered as potential risk genes for MDD. Aberrant genes are known to predispose subjects to depression (31), although the validation of MDD susceptibility genes, including TPH2 and 5-HT2A, its interaction is unclear leading to the need for further investigations of the potential gene-gene interaction effects played by the key regulators of serotonin system in MDD susceptibility.

In the present study, we attempted to verify the relationship of TPH2 variants (rs4570625, rs120074175, and rs11178997) and 5-HT2A variants (rs7997012, rs6311, and rs6313) with MDD by evaluating their single and interaction effects upon MDD susceptibility in a Chinese Han population. The results of the present study not only suggested the important effect of the TPH2 gene upon the risk of MDD, but also provided preliminary evidence that the interaction between TPH2 and 5-HT2A polymorphism variants may influence MDD susceptibility. To the best of our knowledge, this is the first report to investigate TPH2/5-HT2A interaction effects upon the risk of MDD. In addition, our results suggested that the impaired homeostatic regulation of serotonin may predispose individuals to MDD.

In single-locus association analysis, we observed significant differences in genotypic and allelic distribution with the rs11178997 and rs120074175 TPH2 variants. The number of A-allele individuals for rs11178997/rs120074175 was significantly greater in the MDD group than in the control group, which revealed that A-allele carriers of rs11178997 and rs120074175 were more likely to suffer from MDD with a 1.637-fold and a 7.909-fold increased risk, respectively, compared to non-carriers. This result suggested that the TPH2 gene might play a major role in MDD, particularly the rs11178997 TPH2 polymorphism, which had the most independent effect. Previous studies have found that both the rs11178997 (27) and the rs120074175 (8) polymorphism affected the transcriptional activity of TPH2, thereby influencing serotonin production. Papers by Van Den Bogaert et al. and Cichonet al. have, respectively reported an association between rs11178997 and unipolar or bipolar affective disorder (32, 33),while Zhang et al. reported an association of rs120074175 with unipolar major depression (8).

An earlier review suggested that the pathophysiology of depression might result, at least in part, from the direct dysregulation of brain 5-HT2A neurotransmission or indirectly from the dysfunction of other neurotransmitter systems that are under the control of 5-HT2A (34). Some existing studies have attempted to explore the association between the 5-HT2A SNPs and MDD (35–37), but few of these have met with success (38, 39). After Bonferroni correction, our current data showed no association of three 5-HT2A SNPs (rs7997012, rs6311, rs6313) with MDD at either the single-locus or haplotypic level. The inconsistency in our data may have arisen due to sample sizes, different ethnicities, and different definitions of disease; however, the negative results of the single marker analyses do not preclude the fact that the 5-HT2A gene variation has a minor effect upon MDD susceptibility (40). A recent study found that 5-HT2A mRNA levels in the peripheral blood mononuclear cells of MDD patients may have been associated with the severity of depression and the duration of illness in an Iranian population (41).

GMDR analysis can improve the predictive power of genetic association with MDD (42), and has been used to investigate gene-gene interaction in other biological pathways. GMDR is a non-parametric and genetic model for detecting and characterizing non-linear interactions among discrete genetic attributes that are sensitive to the detection of high-order interactions (43). Our GMDR results indicated potential gene-gene interactions between TPH2 and 5-HT2A with significant 2-locus to 6-locus interaction models, which confer a greater susceptibility to MDD. The best TPH2/5-HT2A interaction model was a 3-locus model (TPH2: rs120074175, rs11178997; 5-HT2A: rs7997012) using age and gender as covariates, which had a higher testing accuracy than the multi-locus interaction model of the single TPH2 gene. Identifying interaction between TPH2 and 5-HT2A is likely to contribute to a better understanding of genetic predisposition to MDD. In addition, we also found that both rs11178997 and rs120074175 have greater independent effects in terms of TPH2/5-HT2A interaction, which was far stronger than some other MDD susceptibility genes (44), and there was a strong redundant interaction between these variants, suggesting that TPH2 polymorphism may play vital roles in interaction.

Furthermore, previous research has suggested that the biological interaction between TPH2 and 5-HT2A is involved in the development of MDD. An animal study showed, using a TPH2 decrease-of-function model, gave rise to increased 5-HT2A binding in some brain regions (18). Another animal study observed that long-term activation of the 5-HT2A receptor induced an increase in TPH2 gene expression, TPH2 activity, and serotonin levels (15). These findings suggest that concurrent TPH2 and 5-HT2A variations may develop into long-term abnormal serotonin levels, thus predisposing individuals to MDD.

In interpreting the results of the current study, it is important to consider the following limitations. Firstly, in the current study, the geographic region and ethnic origin were strictly controlled to reduce the potential effects of population stratification; consequently, our data need to be further verified in other ethnicities and geographical regions. Secondly, our sample size was relatively small with only 565 subjects (case: 278, control: 287), although our study still possessed a *post-hoc* power of 0.99 to detect a moderate (0.5) effect size at the 0.05 significance level (two-tailed). Thirdly, SNPs were chosen in this study with more evidence that they are associated with MDD. The interaction of other TPH2/5-HT2A SNPs, which are potentially associated with MDD, was not investigated in the present study. It is therefore necessary to perform further investigations to better understand the role of interaction between TPH2 and 5-HT2A polymorphisms in MDD susceptibility.

## Conclusion

For the first time, this study reports the interaction of serotonin-related genes, TPH2 and 5-HT2A, upon MDD susceptibility in a Chinese Han population. Our data identified an important effect of TPH2 gene variants (rs11178997 and rs120074175) upon the risk of MDD, and suggested that the interaction of TPH2/5-HT2A polymorphism variants confer a greater susceptibility to MDD. The present study also provided preliminary evidence that the interaction of TPH2 and 5-HT2A polymorphism variants may influence the susceptibility to MDD in a Chinese Han population, and suggested that impaired serotonin homeostatic regulation may be a risk factor for MDD.

## Ethics Statement

This study was carried out in accordance with the recommendations of the Ethics Committee of Harbin Medical University with written informed consent from all subjects. All subjects gave written informed consent in accordance with the Declaration of Helsinki. The protocol was approved by the Ethics Committee of Harbin Medical University.

## Author Contributions

JY and YY designed the study. XQ, XY, and ZQ participated in the acquisition of data, which were analyzed by XuZ and JM. JY and XuZ wrote the article. BB, XiZ, and DC critically reviewed it. All authors assisted in the revision process, and gave approval for the final version of the article to be published.

### Conflict of Interest Statement

The authors declare that the research was conducted in the absence of any commercial or financial relationships that could be construed as a potential conflict of interest.

## References

[1] Kessler RC Bromet EJ. The epidemiology of depression across cultures. Annu Rev Public Health. (2013) 34:119–38. 10.1146/annurev-publhealth-031912-114409 23514317 PMC4100461

[2] Avenevoli S Swendsen J He JP Burstein M Merikangas KR. Major depression in the national comorbidity survey-adolescent supplement: prevalence, correlates, and treatment. J Am Acad Child Adolesc Psychiatry. (2015) 54:37–44 e32. 10.1016/j.jaac.2014.10.010 25524788 PMC4408277

[3] Sullivan PF Neale MC Kendler KS. Genetic epidemiology of major depression: review and meta-analysis. Am J Psychiatry. (2000) 157:1552–62. 10.1176/appi.ajp.157.10.1552 11007705

[4] Zubenko GS Zubenko WN Spiker DG Giles DE Kaplan BB. Malignancy of recurrent, early-onset major depression: a family study. Am J Med Genet. (2001) 105:690–9. 10.1002/ajmg.1554 11803516

[5] Andrews PW Bharwani A Lee KR Fox M Thomson JA Jr Is serotonin an upper or a downer? The evolution of the serotonergic system and its role in depression and the antidepressant response. Neurosci Biobehav Rev. (2015) 51:164–88. 10.1016/j.neubiorev.2015.01.018 25625874

[6] Belmaker RH Agam G. Major depressive disorder. N Engl J Med. (2008) 358:55–68. 10.1056/NEJMra073096 18172175

[7] Walther DJ Peter JU Bashammakh S Hortnagl H Voits M Fink H. Synthesis of serotonin by a second tryptophan hydroxylase isoform. Science. (2003) 299:76. 10.1126/science.1078197 12511643

[8] Zhang X Gainetdinov RR Beaulieu JM Sotnikova TD Burch LH Williams RB. Loss-of-function mutation in tryptophan hydroxylase-2 identified in unipolar major depression. Neuron. (2005) 45:11–6. 10.1016/j.neuron.2004.12.014 15629698

[9] Jacobsen JP Siesser WB Sachs BD Peterson S Cools MJ Setola V. Deficient serotonin neurotransmission and depression-like serotonin biomarker alterations in tryptophan hydroxylase 2 (Tph2) loss-of-function mice. Mol Psychiatry. (2012) 17:694–704. 10.1038/mp.2011.50 21537332 PMC3536482

[10] Zill P Baghai TC Zwanzger P Schule C Eser D Rupprecht R. SNP and haplotype analysis of a novel tryptophan hydroxylase isoform (TPH2) gene provide evidence for association with major depression. Mol Psychiatry. (2004) 9:1030–6. 10.1038/sj.mp.4001525 15124006

[11] Gao J Pan Z Jiao Z Li F Zhao G Wei Q. TPH2 gene polymorphisms and major depression–a meta-analysis. PLoS ONE. (2012) 7:e36721. 10.1371/journal.pone.0036721 22693556 PMC3365065

[12] Zhou Z Roy A Lipsky R Kuchipudi K Zhu G Taubman J. Haplotype-based linkage of tryptophan hydroxylase 2 to suicide attempt, major depression, and cerebrospinal fluid 5-hydroxyindoleacetic acid in 4 populations. Arch Gen Psychiatry. (2005) 62:1109–18. 10.1001/archpsyc.62.10.1109 16203956

[13] Angoa-Perez M Kane MJ Briggs DI Herrera-Mundo N Sykes CE Francescutti DM Mice genetically depleted of brain serotonin do not display a depression-like behavioral phenotype. ACS Chem Neurosci. (2014) 5:908–19. 10.1021/cn500096g 25089765 PMC4777283

[14] Hammer C Degenhardt F Priebe L Stutz AM Heilmann S Waszak SM A common microdeletion affecting a hippocampus- and amygdala-specific isoform of tryptophan hydroxylase 2 is not associated with affective disorders. Bipolar Disord. (2014) 16:764–8. 10.1111/bdi.12207 24754353

[15] Naumenko VS Tsybko AS Bazovkina DV Popova NK. Implication of 5-HT2A receptors in the genetic mechanisms of the brain 5-HT system autoregulation. Mol Biol (Mosk). (2012) 46:416–22. 10.1134/S0026893312020100 22888631

[16] Smith RM Papp AC Webb A Ruble CL Munsie LM Nisenbaum LK. Multiple regulatory variants modulate expression of 5-hydroxytryptamine 2A receptors in human cortex. Biol Psychiatry. (2013) 73:546–54. 10.1016/j.biopsych.2012.09.028 23158458 PMC3582836

[17] Pinborg LH Adams KH Yndgaard S Hasselbalch SG Holm S Kristiansen H. [18F]altanserin binding to human 5HT2A receptors is unaltered after citalopram and pindolol challenge. J Cereb Blood Flow Metab. (2004) 24:1037–45. 10.1097/01.WCB.0000126233.08565.E7 15356424

[18] Jorgensen CV Jacobsen JP Caron MG Klein AB Knudsen GM Mikkelsen JD Cerebral 5-HT2A receptor binding, but not mGluR2, is increased in tryptophan hydroxylase 2 decrease-of-function mice. Neurosci Lett. (2013) 555:118–22. 10.1016/j.neulet.2013.08.073 24055299 PMC4323164

[19] Rosel P Arranz B San L Vallejo J Crespo JM Urretavizcaya M Altered 5-HT(2A) binding sites and second messenger inositol trisphosphate (IP(3)) levels in hippocampus but not in frontal cortex from depressed suicide victims. Psychiatry Res. (2000) 99:173–81. 10.1016/S0925-4927(00)00076-7 11068198

[20] Rosel P Arranz B Urretavizcaya M Oros M San L Navarro MA. Altered 5-HT2A and 5-HT4 postsynaptic receptors and their intracellular signalling systems IP3 and cAMP in brains from depressed violent suicide victims. Neuropsychobiology. (2004) 49:189–95. 10.1159/000077365 15118356

[21] Jokela M Lehtimaki T Keltikangas-Jarvinen L. The influence of urban/rural residency on depressive symptoms is moderated by the serotonin receptor 2A gene. Am J Med Genet B Neuropsychiatr Genet. (2007) 144B:918–22. 10.1002/ajmg.b.30555 17510953

[22] Brezo J Bureau A Merette C Jomphe V Barker ED Vitaro F. Differences and similarities in the serotonergic diathesis for suicide attempts and mood disorders: a 22-year longitudinal gene-environment study. Mol Psychiatry. (2010) 15:831–43. 10.1038/mp.2009.19 19381154

[23] Kamata M Suzuki A Yoshida K Takahashi H Higuchi H Otani K. Genetic polymorphisms in the serotonergic system and symptom clusters of major depressive disorder. J Affect Disord. (2011) 135:374–6. 10.1016/j.jad.2011.08.027 21917318

[24] Lopizzo N Bocchio Chiavetto L Cattane N Plazzotta G Tarazi FI Pariante CM. Gene-environment interaction in major depression: focus on experience-dependent biological systems. Front Psychiatry. (2015) 6:68. 10.3389/fpsyt.2015.00068 26005424 PMC4424810

[25] Cardon LR Bell JI. Association study designs for complex diseases. Nat Rev Genet. (2001) 2:91–9. 10.1038/35052543 11253062

[26] Moore JH. A global view of epistasis. Nat Genet. (2005) 37:13–4. 10.1038/ng0105-13 15624016

[27] Scheuch K Lautenschlager M Grohmann M Stahlberg S Kirchheiner J Zill P. Characterization of a functional promoter polymorphism of the human tryptophan hydroxylase 2 gene in serotonergic raphe neurons. Biol Psychiatry. (2007) 62:1288–94. 10.1016/j.biopsych.2007.01.015 17568567

[28] Preuss N Salehi B van der Veen JW Shen J Drevets WC Hodgkinson C. Associations between prefrontal gamma-aminobutyric acid concentration and the tryptophan hydroxylase isoform 2 gene, a panic disorder risk allele in women. Int J Neuropsychopharmacol. (2013) 16:1707–17. 10.1017/S1461145713000254 23552096 PMC4025920

[29] Ruble CL Smith RM Calley J Munsie L Airey DC Gao Y. Genomic structure and expression of the human serotonin 2A receptor gene (HTR2A) locus: identification of novel HTR2A and antisense (HTR2A-AS1) exons. BMC Genet. (2016) 17:16. 10.1186/s12863-015-0325-6 26738766 PMC4702415

[30] Albert PR Benkelfat C. The neurobiology of depression–revisiting the serotonin hypothesis. II Genetic, epigenetic and clinical studies. Philos Trans R Soc Lond B Biol Sci. (2013) 368:20120535. 10.1098/rstb.2012.0535 23440469 PMC3638388

[31] Wurtman RJ. Genes, stress, and depression. Metabolism. (2005) 54(5 Suppl. 1):16–9. 10.1016/j.metabol.2005.01.007 15877307

[32] Van Den Bogaert A Sleegers K De Zutter S Heyrman L Norrback KF Adolfsson R. Association of brain-specific tryptophan hydroxylase, TPH2, with unipolar and bipolar disorder in a Northern Swedish, isolated population. Arch Gen Psychiatry. (2006) 63:1103–10. 10.1001/archpsyc.63.10.1103 17015812

[33] Cichon S Winge I Mattheisen M Georgi A Karpushova A Freudenberg J. Brain-specific tryptophan hydroxylase 2 (TPH2): a functional Pro206Ser substitution and variation in the 5'-region are associated with bipolar affective disorder. Hum Mol Genet. (2008) 17:87–97. 10.1093/hmg/ddm286 17905754

[34] Guiard BP Di Giovanni G. Central serotonin-2A (5-HT2A) receptor dysfunction in depression and epilepsy: the missing link? Front Pharmacol. (2015) 6:46. 10.3389/fphar.2015.00046 25852551 PMC4362472

[35] Gu L Long J Yan Y Chen Q Pan R Xie X HTR2A-1438A/G polymorphism influences the risk of schizophrenia but not bipolar disorder or major depressive disorder: a meta-analysis. J Neurosci Res. (2013) 91:623–33. 10.1002/jnr.23180 23404241

[36] Jin C Xu W Yuan J Wang G Cheng Z. Meta-analysis of association between the−1438A/G (rs6311) polymorphism of the serotonin 2A receptor gene and major depressive disorder. Neurol Res. (2013) 35:7–14. 10.1179/1743132812Y.0000000111 23317793

[37] Tan J Chen S Su L Long J Xie J Shen T. Association of the T102C polymorphism in the HTR2A gene with major depressive disorder, bipolar disorder, and schizophrenia. Am J Med Genet B Neuropsychiatr Genet. (2014) 165B:438–55. 10.1002/ajmg.b.32248 24962835

[38] Petit AC Quesseveur G Gressier F Colle R David DJ Gardier AM. Converging translational evidence for the involvement of the serotonin 2A receptor gene in major depressive disorder. Prog Neuropsychopharmacol Biol Psychiatry. (2014) 54:76–82. 10.1016/j.pnpbp.2014.04.013 24801750

[39] Cao S Li H Lou L Xie Z Zhao X Pang J. Association study between 5-HT2A and NET gene polymorphisms and recurrent major depression disorder in Chinese Han population. Pak J Pharm Sci. (2015) 28:1101–8. 26051731

[40] Lin E Chen PS Chang HH Gean PW Tsai HC Yang YK. Interaction of serotonin-related genes affects short-term antidepressant response in major depressive disorder. Prog Neuropsychopharmacol Biol Psychiatry. (2009) 33:1167–72. 10.1016/j.pnpbp.2009.06.015 19560507

[41] Amidfar M Kim YK Colic L Arbabi M Mobaraki G Hassanzadeh G. Increased levels of 5HT2A receptor mRNA expression in peripheral blood mononuclear cells of patients with major depression: correlations with severity and duration of illness. Nord J Psychiatry. (2017) 71:1–10. 10.1080/08039488.2016.1276624 28125323

[42] Roetker NS Yonker JA Lee C Chang V Basson JJ Roan CL. Multigene interactions and the prediction of depression in the Wisconsin Longitudinal Study. BMJ Open. (2012) 2:e000944. 10.1136/bmjopen-2012-000944 22761283 PMC3391375

[43] Ma J Xiao H Yang Y Cao D Wang L Yang X. Interaction of tryptophan hydroxylase 2 gene and life events in susceptibility to major depression in a Chinese Han population. J Affect Disord. (2015) 188:304–9. 10.1016/j.jad.2015.07.041 26386440

[44] Li Z Zhang Y Wang Z Chen J Fan J Guan Y. The role of BDNF, NTRK2 gene and their interaction in development of treatment-resistant depression: data from multicenter, prospective, longitudinal clinic practice. J Psychiatr Res. (2013) 47:8–14. 10.1016/j.jpsychires.2012.10.003 23137999 PMC3584686

